# Toll-like receptor 4 (TLR4) deficient mice are protected from adipose tissue inflammation in aging

**DOI:** 10.18632/aging.101288

**Published:** 2017-09-07

**Authors:** Amiya K. Ghosh, Martin O'Brien, Theresa Mau, Raymond Yung

**Affiliations:** ^1^ Division of Geriatric and Palliative Medicine, Department of Internal Medicine, University of Michigan, Ann Arbor, MI 48109, USA; ^2^ Graduate Program in Immunology, University of Michigan, Ann Arbor, MI 48109, USA; ^3^ Geriatric Research, Education and Clinical Care Center (GRECC), VA Ann Arbor Health System, Ann Arbor, MI 48109, USA

**Keywords:** aging, Inflammation, obesity

## Abstract

Adipose tissue (AT) inflammation is a central mechanism for metabolic dysfunction in both diet-induced obesity and age-associated obesity. Studies in diet-induced obesity have characterized the role of Fetuin A (Fet A) in Free Fatty Acids (FFA)-mediated TLR4 activation and adipose tissue inflammation. However, the role of Fet A & TLR4 in aging-related adipose tissue inflammation is unknown. In the current study, analysis of epidymymal fat pads of C57/Bl6 male mice, we found that, in contrast to data from diet-induced obesity models, adipose tissue from aged mice have normal Fet A and TLR4 expression. Interestingly, aged TLR4-deficient mice have diminished adipose tissue inflammation compared to normal controls. We further demonstrated that reduced AT inflammation in old TLR4-deficient mice is linked to impaired ER stress, augmented autophagy activity, and diminished senescence phenomenon. Importantly, old TLR4-deficient mice have improved glucose tolerance compared to age-matched wild type mice, suggesting that the observed reduced AT inflammation in aged TLR4-deficient mice has important physiological consequences. Taken together, our present study establishes novel aspect of aging-associated AT inflammation that is distinct from diet-induced AT inflammation. Our results also provide strong evidence that TLR4 plays a significant role in promoting aging adipose tissue inflammation.

## INTRODUCTION

Adipose tissue inflammation has become widely accepted as a major contributor to metabolic dysfunction and disorders [1, 2]. Previous studies on diet induced obesity (DIO) mice have shown that adipose tissue is primed for inflammatory changes prior to other metabolic organs [3]. There is a plethora of research investigating factors in obese adipose tissue inflammation to identify valuable therapeutic targets for metabolic dysfunction. However, much less is under-stood about aging adipose tissue inflammation and dysfunction. A better understanding of the cellular and molecular mechanisms of adipose tissue inflammation in aging will be crucial in the development of therapeutics for metabolic diseases beyond cases of diet-induced adipose tissue inflammation and insulin resistance.

Both age-related adiposity and diet-induced obesity are characterized by immune cell infiltration and a sustained inflammatory cycle. Among these various immune cells, adipose tissue macrophage (ATM) accumulation, proliferation, and polarization are major contributors to adipose tissue inflammation and dysfunction [4, 5]. Interestingly, recent studies suggest that changes in preadipocyte function during aging also lead to dysfunctional adipose tissue, eventually progressing to chronic inflammation [6]. These changes include reduced preadipocyte replication, decreased adipogenesis, increased lipid toxicity, increased pro-inflammatory cytokines, chemokines, extracellular matrix (ECM)-modifying proteases, and stress response elements. Our group have recently shown that elevated endoplasmic reticulum (ER) stress response in aging also contributes to greater inflammatory responses [7], in part due to compromised autophagy activity in the aging adipose tissue [8]. Recent studies have also indicated that with aging there is increased accumulation of senescent cells in many organs including fat depots, which contributes to aging pathologies [9]. Moreover, removal of senescent cells by senolytic drugs rescue adipose tissue from the aging phenotype [10, 11]. However, the detailed molecular mechanisms that lead to increased inflammation in aging adipose tissue are poorly defined.

During the last decade, major advances were made in identifying the molecular mechanisms by which lipid-derived products promote inflammation in different cell types [12]. These lipid derived products, which include ceramides and Di-Acyle glycerol (DAG), induce insulin resistance via de-phosphorylation of protein kinase B (PKB) and phosphorylation of a serine residue of insulin receptor substrate 1 (IRS-1) [13, 14]. Another lipid-derived product, non-esterified fatty acids (NEFA), elevates tissue inflammation through inter-action with the pattern recognition receptor Toll-like receptor 4 (TLR4) via its endogenous ligand Fetuin-A (Fet A), a liver derived glycoprotein [15]. Fet A is considered a biomarker of chronic inflammation due to its ability to stimulate the production of inflammatory mediators from both adipocytes and macrophages [16]. Studies examining the role of Fet A in obesity have shown that free fatty acid (FFA)-induced insulin resistance is dependent on the presence of both Fet A and TLR4, where Fet A serves as an adaptor for FFA to stimulate TLR4 signaling that results in the release of pro-inflammatory cytokines through the TLR4-NF kB pathway [15]. These findings were further supported by studies that showed TLR4^-/-^ mice fed with high fat diet (HFD) have alternative macrophage polarization, reduced AT inflammation, and decreased hepatic steatosis [17-19]. Interestingly, Fet-A null mice were also protected against obesity and insulin resistance with aging [20].

The involvement of Fet A-mediated activation of TLR4 pathway in adipose tissue inflammation in diet-induced obesity is well explored. However, the role of this pathway in age-associated adipose tissue inflammation is unknown. We undertook this study to test the hypothesis that age-related adipose tissue inflammation is dependent on the Fet A-mediated TLR4 signaling pathway. We first evaluated the expression of *Tlr4* and *Fet A* gene products in adipose tissue, liver, and plasma samples derived from young and old mice. We then exploited the TLR4-deficient mice to investigate the role of TLR4 in age-associated adipose tissue inflammation, ER stress response, autophagy activity, cellular senescence, and metabolic status (glucose tolerance).

## RESULTS

### Adipose tissue expression of TLR4 and Fet A are elevated in DIO but not in aging

**Figure 1:**
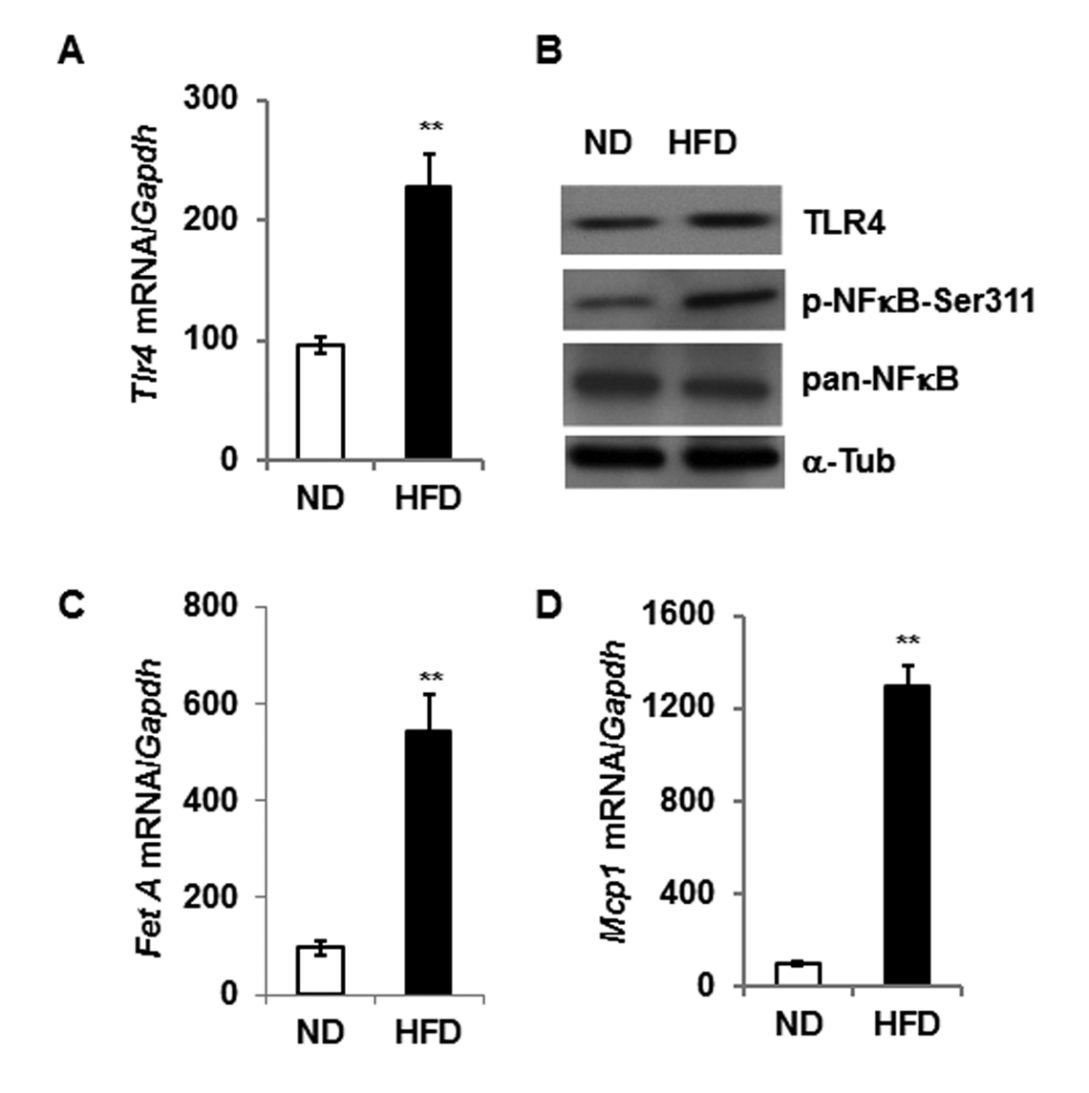
High fat diet induced expression of Tlr4, Fet A and Mcp1 in the adipose tissue *Tlr4* gene expression pattern at mRNA (**A**) and protein (**B**) level in adipose tissue of 5 m old mice fed with either normal diet (ND) or high fat diet (HFD) for 16 weeks. mRNA expression of Fet A is presented in (**C**) and Mcp1 in (**D**). Data represented in bar diagrams are mean ± SD value of relative mRNA expression from three independent experiments where total RNA was extracted from gonadal fat pads of ND (n=5) and HFD (n=5) mice. Proteins levels were analyzed by western blotting. Data presented here are representative image of three independent experiments. The significance levels **p<0.001 were analyzed by unpaired Student's t-test using means and SDs.

**Figure 2:**
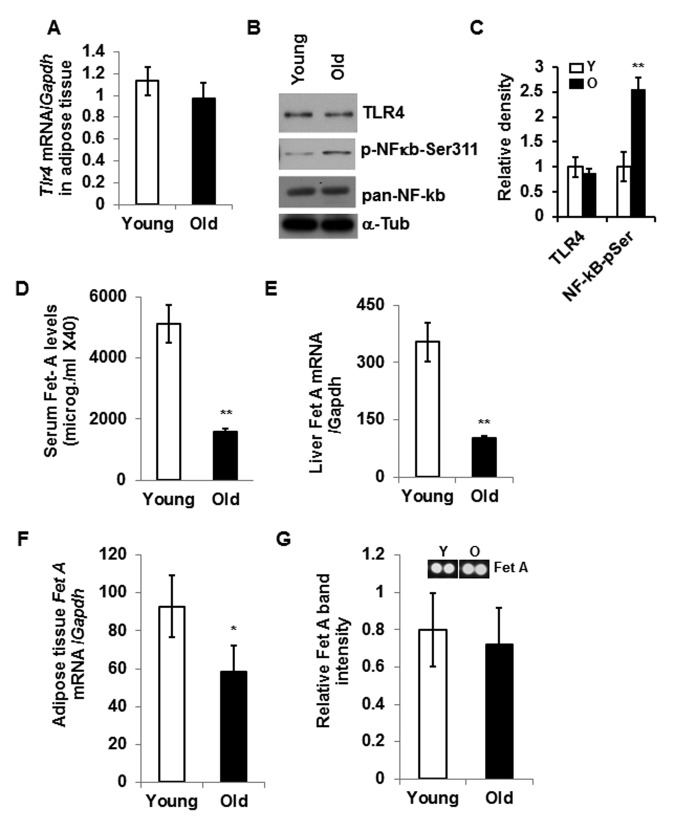
Aging associated adipose tissue inflammation is not correlated with the expression of TLR4 or its endogenous ligand, Fet A Relative expression of *Tlr4* gene in the adipose tissue of young or old mice at mRNA (**A**) and protein (**B**) levels. NF-κb activation was determined by western blotting using NF-κB-phospho-serine311 antibody (**B**). The relative density is expressed in (**C**). (**D**) Serum Fet A levels in young and old mice. (**E**) Relative mRNA expression of Fet A in the liver. mRNA expression of Fet A (**F**) and protein levels (**G**) in the adipose tissue of young and old mice. Data represented in bar diagrams are mean ± SD value of relative mRNA expression from three independent experiments where total RNA was extracted from gonadal fat pads of young (n=5) and old (n=5) mice and used as a template for one-step qRT-PCR reaction. Protein expressions were determined by western blotting of adipose tissue lysates from young (n=5) and old (n=5) mice. Data presented here are representative image of three independent experiments. The significance levels *p<0.05, **p<0.001 were analyzed by unpaired Student's t-test using means and SDs.

Consistent with previous reports [15, 21], our present analysis indicates that DIO increases the expression of both TLR4 and Fet A, and contributes to adipose tissue inflammation as indicated by higher mRNA expression of *Mcp1* (Fig.1). We then analyzed and compared the expression of *Tlr4* in epididymal adipose tissues from young and old mice and observed no significant differences at either mRNA or protein levels (Fig. 2A and 2B). However, there is enhanced activation of NF-κB (elevated serine-311 phosphorylation) in adipose tissue lysates of old mice compared to young (Fig. 2C). Since Fet A has been reported to be an adaptor of FFA and a ligand of TLR4 in DIO, we analyzed the serum Fet A levels in both young and old mice. Surprisingly, serum levels of Fet A in old mice were significantly lower than those in young mice (Fig. 2D). Since liver is the main source of Fet A, we examined *Fet A* gene expression in liver of young and old mice. Our analyses indicated diminished *Fet A* mRNA levels compared to the young cohort (Fig. 2E). We then performed mRNA analysis on adipose tissue samples from young and old mice and the results indicated that *Fet A* mRNA level was also diminished in old mice (Fig. 2F). Finally, we did not observe significant difference in Fet A protein expression between young and old adipose tissue lysates (Fig.2G). We also analyzed mRNA expression of *fibronectin* and *Tenacin C1*, which are reported to serve as endogenous ligands for TLR4 to promote inflammation in HFD-induced obesity [22] and in arthritis model [23]. We observed diminished expression of both *fibronectin* and *Tenacin C1* in the aging adipose tissue (Fig. S1). Our results therefore indicate that, contrary to DIO, adipose tissue inflammation in aging is not dependent on the Fet A-mediated TLR4 activation pathway.

### Reduced adipose tissue inflammation in TLR4 deficient mice

**Figure 3:**
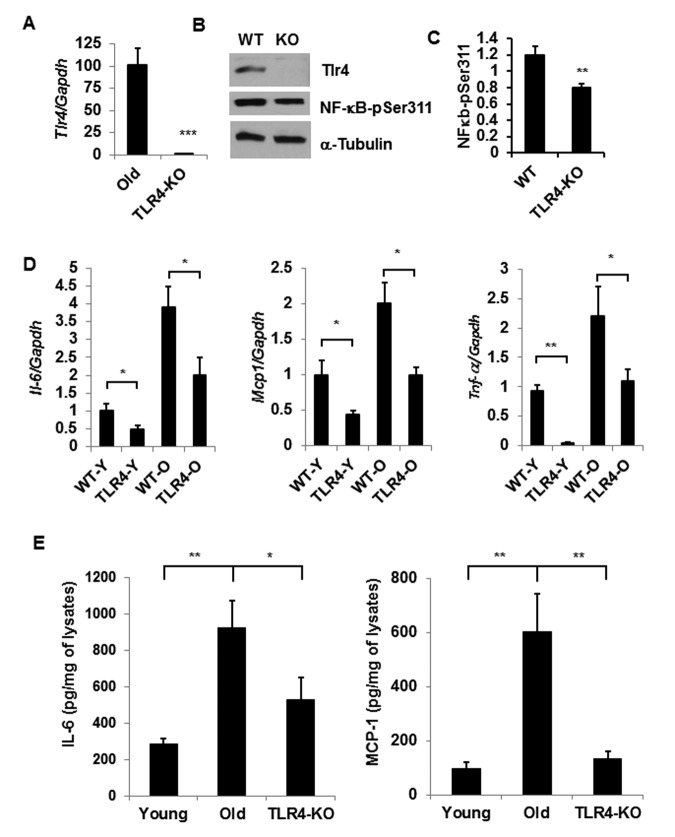
TLR-4 mutant old mice are protected from adipose tissue inflammation Expression of *Tlr4* gene products: mRNA (**A**) and protein (**B**) levels together with NF-κB activation by using a phospho-NF-κB p65 pSer311 antibody in western blotting analysis. The relative protein density is expressed in (**C**). (**D**) Expression profile of pro-inflammatory cytokine genes (*Il-6, Mcp1 and Tnf-a*) in the adipose tissue of young or old of both WT and TLR4 deficient mice. (**E**) The protein product of IL-6 and MCP1 in the SVF lysates derived from the adipose tissues of young WT and old WT or old TLR4 deficient mice. Data represented in bar diagrams are mean ± SD value of relative mRNA expression from three independent experiments where total RNA was extracted from gonadal fat pads of young (n=5) and old (n=5) mice. Data presented here are representative images of three independent experiments. The significance levels *p<0.05, **p<0.001 were analyzed by unpaired Student's t-test using means and SDs.

To understand the role of TLR4 in aging adipose tissue inflammation, we conducted studies using a TLR4 deficient (*Tlr4^Lps-del^)* mouse model. We first evaluated the expression of *Tlr4* in wild type (C57BL/6) and *Tlr4^Lps-del/^*/*TLR4-KO* mice and validated that TLR4 expression was absent in the KO mice (Fig. 3A). We observed diminished p-NFκB expression in the aging adipose tissue of TLR4-KO mice compared to the WT age-matched controls (Fig. 3B and 3C). Our analyses indicated that the expressions of three major pro-inflammatory cytokines (*Il-6, Mcp1* and *Tnf-ɑ*) in the adipose tissue were significantly reduced in old TLR4-KO mice compared to the old wild type mice (Fig.3D). Protein analysis on adipose tissue lysates also indicated decreased levels of IL-6 and MCP1 in old TLR4-KO compared to age-matched WT mice (Fig.3E). Of note, we were unable to detect TNF-ɑ in the adipose tissue lysates by ELISA method. This data suggests that TLR4 contributed to aging adipose tissue inflammation in a Fet A-independent manner.

### ER stress response and autophagy activity in the adipose tissue of TLR4 deficient old mice

**Figure 4:**
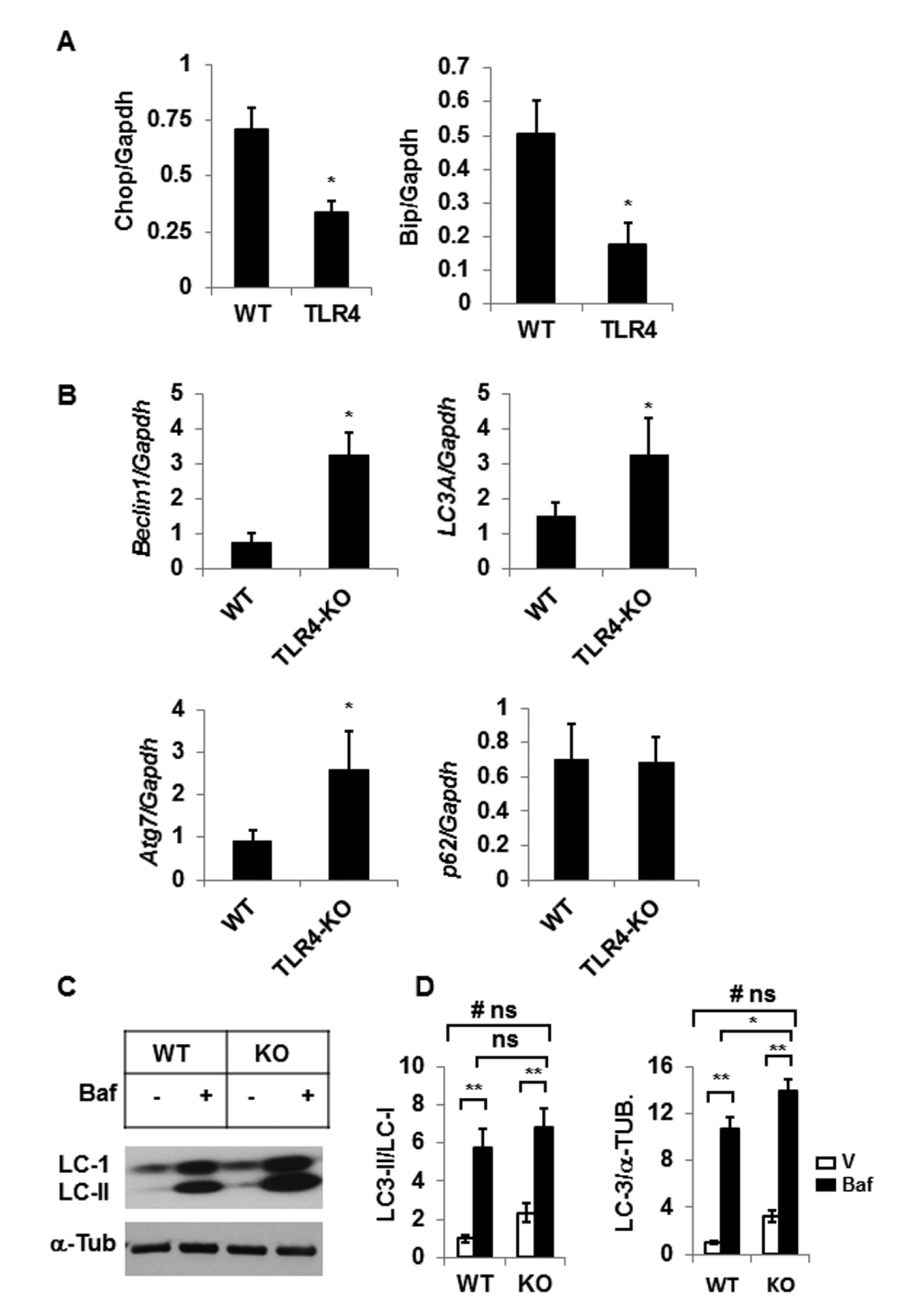
TLR4-KO old mice are protected from adipose tissue inflammation and ER stress Relative mRNA expressions of ER stress response genes (**A**) and autophagy genes (**B**) in adipose tissue of old (20 m) WT and TLR4 deficient mice were analyzed by qRT-PCR. Data represented in bar diagrams are mean ± SD value of relative mRNA expression from three independent experiments where total RNA was extracted from the adipose tissue of WT (n=5) and TLR4 deficient (n=5) mice and used as a template for one-step qRT-PCR reaction. (**C**) Western blot analysis of LC3- II/I from SVFs lysates isolated from WT (n=5) and TLR4-KO (n=5) and pooled and treated with either vehicle (DMSO) or Baf (10 nM) for 18h in culture. The density of protein bands from three independent experiments were normalized with ɑ-tubulin and plotted (**D**). Values were presented as mean ± SD of three independent experiments. Significance of difference between means was determined by Student's t-test and indicated by ^*^p<0.05 and ^**^p<0.01. Symbol ^#^ indicated the significance level (p<0.05) of two-way ANOVA analysis for the interaction between treatment (vehicle and Baf) and age factor (WT vs. KO). No significant differences are indicated by ns.

Our recent studies have demonstrated that impaired autophagy leads to elevated ER stress in aging adipose tissue [8]. We sought to determine if TLR4 deficient mice were protected from the elevated ER stress response and reduced autophagy activity in aging. Consistent with our previous reports [7], expression of ER stress response genes *Chop* and *Bip* are reduced in the adipose tissue of TLR4 deficient mice compared to the age-matched old wild type mice (Fig. 4A). Additionally, expression levels of autophagy genes (*Beclin-1, Atg7, Lc3a*), except *p62*, are also increased in the mutant mice (Fig. 4B). To examine the impact of TLR4 deficiency on autophagy activity, we cultured SVFs (stromovascular fraction) from the adipose tissue and autophagy efficiency is measured by accumulation of LC3II/LC-I following bafilomycin (autophagy blocker) treatment. As expected, autophagy activity was enhanced in the SVFs of TLR4-KO mice compared to the WT mice (Fig. 4C and 4D). However, no significant interaction between the variable (WT vs KO) or treatment (V vs Baf) was observed.

### Reduced expression of senescence-associated markers in the adipose tissue of TLR4 deficient old mice

**Figure 5:**
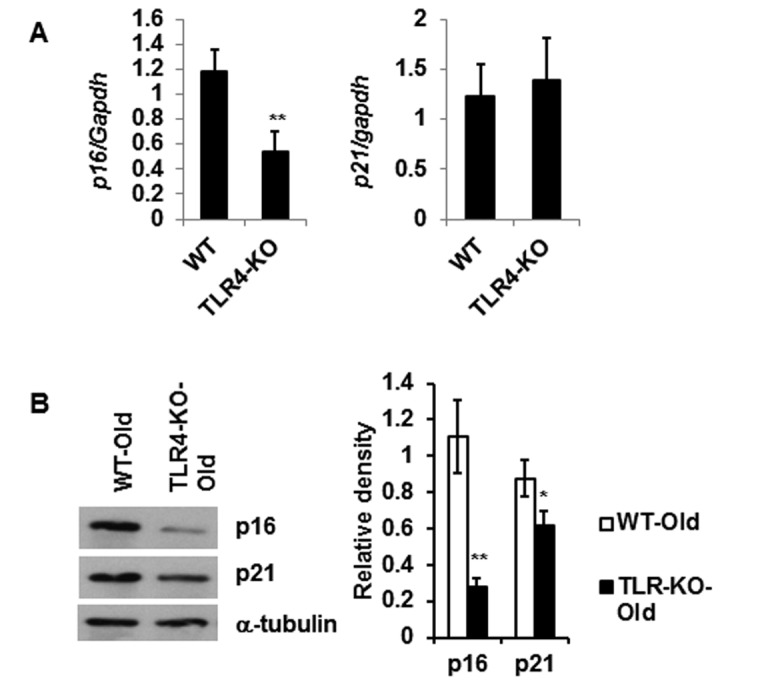
Reduced expression level of senescence associated genes *p16* and *p21* in the adipose tissue of TLR4 deficient old mice Relative mRNA expression of senescence associated genes (**A**) and their protein products (**B**) in adipose tissue of WT and TLR4 deficient old mice (20 m). The relative density of protein bands of p16 and p21 are expressed in bar diagram (**B**, right). Data represented in bar diagrams are mean ± SD value of three independent experiments. Significance of difference between means was determined by Student's t-test and indicated by ^*^ *p*<0.05 and ^**^p<0.01.

With reduced IL-6 and MCP-1 production in the TLR-4 KO mice (Fig. 3E), we next determined if TLR4 KO is associated with change in the expression of senescence-associated genes. We observed reduced mRNA expression of *p16* gene and no difference in the expression of *p21* gene (Fig. 5A) in adipose tissue from TLR4-KO mice compared to age-matched WT old mice. However, protein expression of both p16 and p21 were significantly diminished in the TLR4 deficient adipose tissue lysates (Fig. 5B). These results indicate that aging TLR4 deficient mice are protected from adipose tissue senescence.

### TLR4 KO mice have improved glucose tolerance

**Figure 6:**
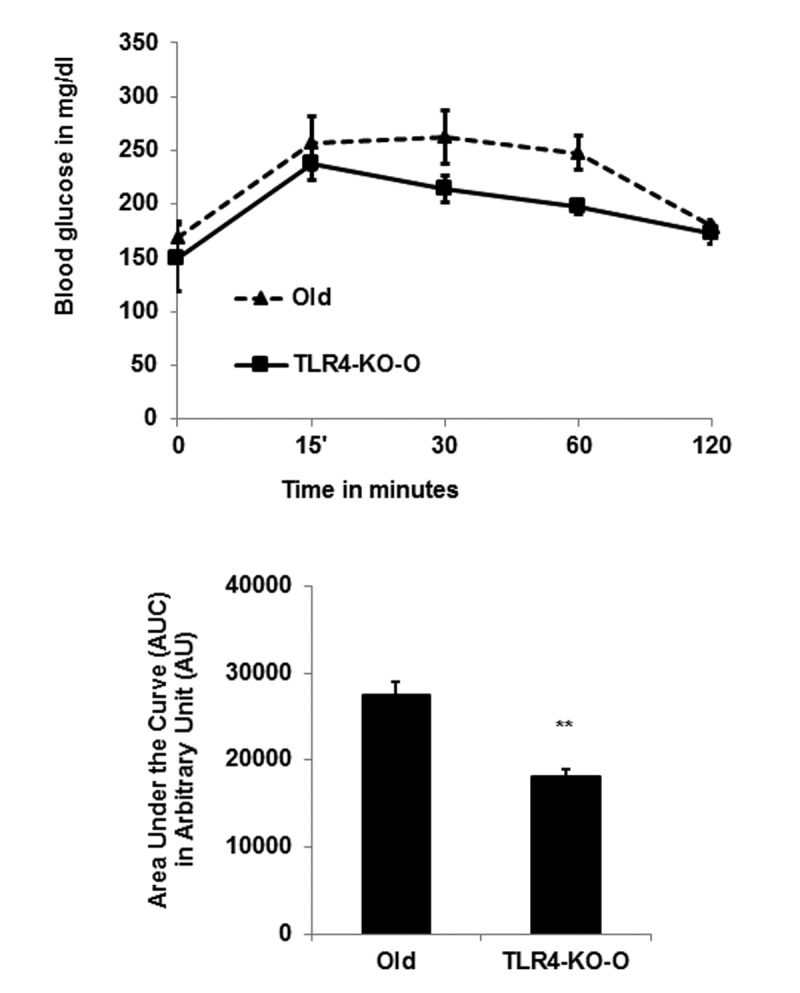
TLR4 deficient mice are more efficient in IPGTT (**A**) Time course of blood glucose concentration following intraperitoneal glucose load in old WT and in TLR4 deficient mice. The area under the curve (AUC) were calculated using the trapezoidal formula and plotted in the bar diagram (**B**). Data represented in bar diagrams are mean ± SD of three independent experiments with WT and TLR4-KO mice (n=4). Significance of difference between means was determined by Student's t-test and indicated ^**^p<0.01.

To determine glucose tolerance, we performed an intraperitoneal glucose tolerance test (IPGTT) on both WT and TLR4 deficient old mice. We found that TLR4 deficient old mice have improved glucose tolerance compared to the age-matched WT mice (Fig. 6A and 6B). The area under the curve (AUC) of TLR4-KO mice is significantly smaller than that of the WT mice, consistent with the idea that TLR4 deficiency promotes glucose tolerance in part through the observed reduced inflammation in visceral adipose tissue.

## DISCUSSION

Chronic low-grade inflammation in adipose tissue contributes to the development of metabolic diseases, including insulin resistance in aging [6, 24]. To understand the mechanism of adipose tissue inflammation, many research groups reported activation of TLR4 receptors by lipids in the context of obesity [1, 15, 25, 26]. Data from obese animals on adipose tissue inflammation have demonstrated that Fet A serves as an adapter for FFA in the activation of the TLR4 pathway [15]. Intriguingly, both Fet A and TLR4 expressions are elevated in the adipose tissue of DIO mouse models, and adipose tissue Fet A serves as a chemoattractant for macrophage migration and polarization [15, 21]. Contrary to the DIO models, we demonstrate here that Fet A level is diminished not only in adipose tissue but also in the serum and liver of old mice (Fig. 2). On the other hand, adipose tissue TLR4 expression remains unchanged between young and old mice. These data indicate that, unlike DIO, the mechanism of aging-associated adipose tissue inflammation is independent of the expression of Fet A or TLR4. To understand TLR4-mediated adipose tissue inflammation in the context of aging, we analyzed the adipose tissue of young and old TLR4 deficient mice, and observed that TLR4 deficiency protects adipose tissue inflammation in old mice (Fig. 3B), as indicated by reduced protein expressions of IL-6 and MCP-1 in the adipose tissue lysates (Fig. 3C).

We have demonstrated previously that elevated endoplasmic reticulum (ER) stress response in aging adipose tissue also contributes to greater inflammatory responses [7] as a result of age-related compromised autophagy activity [8]. The reciprocal interaction between autophagy dysfunction and ER stress has also been investigated in human adipose tissue of type 2 diabetes mellitus (T2DM) [27]. We asked whether TLR4 deficiency protects adipose tissue inflammation in aging through the ER stress response pathway and/or altered autophagy function. We observed diminished ER stress response and elevated expression of autophagy-associated genes in the gonadal adipose tissue of TLR4 deficient mice (Fig. 4). Our data are consistent with the observation that TLR4 deficient mice are also protected from ER stress response in diet-induced obesity [28].

Recent studies have indicated that aging promotes the accumulation of senescent (SEN) cell burden in the VAT, which can also lead to inflammation. This is further supported by the findings of reduced SEN burden in long-lived mouse models [29], and improved health span by clearing SEN cells by drugs (senolytics) in mouse models [9, 11, 30]. Our analyses indicate that expressions of senescence-associated genes (*p16* and *p21*) were diminished in the VAT of TLR4 deficient mice, which may then lead to a reduction of the chronic low-grade inflammation observed in aging (Fig. 5). Finally, we demonstrate that TLR4 deficient old mice have improved glucose tolerance compared to the age-matched WT mice (Fig. 5). This was consistent with the observation that TLR4 deficiency protects the animals from glucose intolerance induced by HFD [28].

Taken together, our Fet A and TLR4 expression data in aging adipose tissue inflammation are in contrast to those seen in DIO models. We have shown that TLR4 deficiency reduces age-associated adipose tissue inflammation by reducing ER stress, augmenting autophagy function, and by reducing senescence cell burden. Our study indicates an important aspect of aging-associated adipose tissue inflammation that is distinct from diet-induced obesity. This study provides strong evidence that TLR4 plays a significant role in promoting aging adipose tissue inflammation. However, we were unable to define the endogenous ligand for TLR4 activation. We have tested other known endo-genous TLR4 ligands, including *Tenacin C1* and *fibronectin*, and they both have diminished expression in the aging adipose tissue (Fig. S1).

The underlying mechanism for TLR4 activation in aging adipose tissue is incompletely understood. Recent progress in the field might explain TLR4 signaling and its regulation as follows:

### 

#### a) Fet A independent activation of TLR4 by lipids

It has been demonstrated previously that lipids such as ceramide and sphingomyelin are more abundant in the aging adipose tissue [31]. In addition, these lipids operate through TLR4 as specific inhibitors (myriocin or Me-SM) of ceramide synthesis pathway both in young or old adipose tissue block the productions of both IL-6 and TNF-ɑ upon LPS treatment.

#### b) Endogenous activation of TLR4 via alarmin HMGB1 (high mobility group box1)

With the accumulation of SEN cells in the aging adipose tissue, there is increased abundance of high mobility group box 1 (HMGB1). It has been demonstrated that extracellular HMGB1 acts as a ligand for TLR4 to produce IL-6, and this effect can be blocked with HMGB1 antibody or TLR4 inhibition in a fibroblast culture system [32]. Interestingly, the circulatory HMGB1 is also elevated in aged mice [32].

#### c) Role of Cbl-b in TLR4 attenuation in macrophages

It has been reported that Casitas B-cell lymphoma gene (Cbl-b) plays a role in aging associated insulin resistance. The HFD caused increased inflammation of adipose tissue in the Cbl^-/-^ mice compared to Cbl^+/+^ mice. This effect is due to Cbl-b suppression of the saturated FA-induced TLR4 signaling by ubiquitination and degradation of TLR4 in macrophage cell line [33].

In summary, we have demonstrated a Fet A-independent role of TLR4 in promoting adipose tissue inflammation in aging adipose tissue. It is likely that TLR4 activation is regulated via one or more of the mechanisms described earlier in promoting aging AT inflammation, but the concept will need further invest-tigation. Interestingly, the current study distinguishes aging adipose from DIO-associated inflammation in the context of TLR4 ligand Fet A, although both are mediated through TLR4 activation.

## MATERIALS AND METHODS

### Mice

Young (4-6 months) and old (18–22 months) C57BL/6 male mice were obtained from the National Institutes of Aging (NIA) aged rodent colonies, Harlan Sprague Dawley (Indianapolis, IN). TLR4 mutant male mice were procured from Jackson Laboratories (stock# 007227). The *Tlr4^Lps-del^* spontaneous mutation corresponds to a 74723 base pair deletion that completely removes the *Tlr4* coding sequence. No mRNA or protein is expressed, and homozygous mutants exhibit a defective response to LPS stimulation.

High fat diet (HFD) regimen: 6-week-old male mice were maintained on normal chow diet (4.7% fat) or on HFD (40% fat) from ENVIGO (Teklad rodent diet) for 16 weeks. All mice were euthanized at 22 weeks of age for analysis.

All mice were maintained in a pathogen-free environment provided by the Unit for Laboratory Animal Medicine (ULAM) at the University of Michigan (Ann Arbor, MI). The University of Michigan University Committee on Use and Care of Animals (UCUCA) has approved all animal experimental protocols in the current study.

### Reagents

Bafilomycin A1 (Baf), Collagenase D, D-glucose were obtained from Sigma-Aldrich (St. Louis, MO). All the chemicals were dissolved in the appropriate media solution or in dimethyl sulfoxide (DMSO) as per manufacturer's instructions and used at the indicated concentrations.

### Isolation of adipose tissue

Epididymal adipose tissues were collected from the mice after euthanasia using standard procedure as described previously [7, 8].

### RNA extraction and real-time RT-PCR

Total RNA was isolated from 100mg of adipose tissue using Qiagen lipid isolation kit and Qiazol. mRNA fold expression changes were estimated by qRT-PCR procedures as previously described [7].

### Western blotting

300mg of AT was placed in 500μL of RIPA buffer supplemented with protease and phosphatase inhibitors and subsequently sonicated to produce cell lysates for western blotting analysis. Standard western blotting techniques were performed for protein analyses as described in previous studies [7, 8]. In brief, 50μg total proteins were separated on SDS-PAGE. The gel was transferred to a PVDF membrane, blocked with Superblock (Thermo Scientific) solution for 30 minutes and incubated with appropriate antibody overnight followed by HRP-conjugated secondary antibody in 5% non-fat dry milk in Tris-buffered saline for blotting and exposure of the membrane to a photographic film.

### Enzyme-Linked Immunosorbent Assay (ELISA)

Quantitation of Fet A in the serum, IL-6, and MCP-1 in the adipose tissue lysates were performed using respective ELISA kits (R & D Systems).

### Intraperitoneal Glucose Tolerance Test (IPGTT)

Mice were fasted overnight for approximately 16 hrs by transferring to clean cages. Mice were weighed to calculate and record the volume of 20% glucose solution required (2g of glucose/kg body mass) for IP injections: volume of IP glucose injection (μl) =10 X body weight (g). Mice were restrained with an approved restrainer device with the tail exposed to score the tip of the tail with a sterilized scalpel blade. The first drop of blood was discarded and a small drop of blood is placed on the test strip of an animal blood glucose meter (Abbott Alpha TRAK 2 Blood Glucose Monitoring System) to record the fasting glucose level (t=0). Appropriate amounts of glucose were injected into the peritoneum as previously determined. The blood glucose levels were measured at 15, 30, 60 and 120 minutes following glucose injections and recorded. At the end of the experimental session, mice were placed in a clean cage with food and water.

## SUPPLEMENTARY MATERIAL FIGURE


