# Supplementary material for: Toll-like receptor 4 (TLR4) deficient mice are protected from adipose tissue inflammation in aging
Source: Aging (Albany NY). 2017 Sep 7;9(9):1971–82. doi: 10.18632/aging.101288 (PMC5636669; doi:10.18632/aging.101288)
Supplement: Supplementary file 1 [file aging-09-1971-s001.pdf]

SUPPLEMENTARY MATERIAL

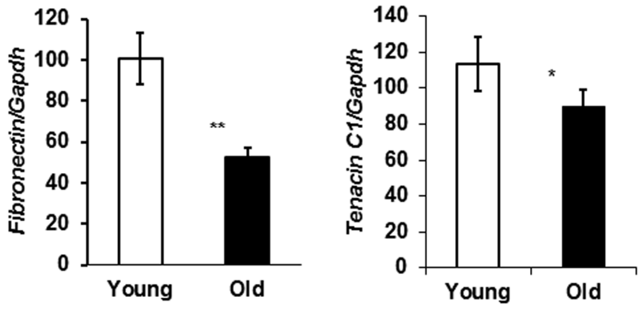

Figure S1. Expression of Fibrinectin and Tenacin1 gene in the yound and old adipose tissue. Data represented in bar diagrams are mean + SD value of relative mRNA expression from three independent experiments where total PNA was extracted from gonadal fat pads of young (n=5) and old (n=5) mice. Significance of difference (P<0.05 or P<0.01) between the means are determined by paired t test.
